# Nutrition Strategies for the Preterm Infant with Bronchopulmonary Dysplasia

**DOI:** 10.3390/nu17213472

**Published:** 2025-11-04

**Authors:** Gabriela S. Trindade, Bianca C. Benincasa, Guilherme S. Procianoy, Rita C. Silveira, Renato S. Procianoy

**Affiliations:** 1 Department of Pediatrics, Newborn Section, Hospital de Clínicas de Porto Alegre, Universidade Federal do Rio Grande do Sul, Porto Alegre 90010-150, RS, Brazil; gabs.trindade@gmail.com (G.S.T.); bcbenincasa@gmail.com (B.C.B.); drarita.c.s@gmail.com (R.C.S.); 2 Medical School, Universidade Federal de Ciências da Saúde de Porto Alegre, Porto Alegre 90050-170, RS, Brazil

**Keywords:** preterm infant, nutrition, extrauterine growth, lung injury, bronchopulmonary dysplasia

## Abstract

**Background/Objectives:** Bronchopulmonary dysplasia (BPD) is a common chronic complication of prematurity, associated with significant morbidity. Nutrition is a key modifiable factor influencing lung growth, repair, and overall development. This review summarizes current evidence on nutritional strategies for BPD prevention and management. **Methods: Narrative review** was conducted with literature search in major databases using relevant keywords. **Results:** Early nutritional deficits are strongly associated with BPD. Higher early protein (3.5–4 g/kg/day) and energy intake (>60 kcal/kg/day in the first week, with progressive increases) reduce ventilator dependence. Lipids are essential to achieve caloric goals. Fluid restriction may reduce BPD risk but often results in undernutrition. Nutrient density, rather than fluid volume, is critical. Enteral nutrition, particularly mother’s own milk, consistently reduces BPD risk, whereas formula feeding is linked to higher BPD incidence. In established BPD, nutritional requirements are substantially increased. Feeding is frequently complicated by fluid restriction, gastroesophageal reflux, and poor oral coordination. Management strategies include higher energy intake (130–150 kcal/kg/day), adequate protein provision (3.5–4 g/kg/day), and careful use of lipid-based energy sources. Fortified human milk or enriched preterm formulas are essential, with individualized fortification improving growth. Micronutrient support is critical, and long-term follow-up is required, as post-discharge growth remains vulnerable and predicts later outcomes. **Conclusions:** Nutritional strategies to mitigate BPD should focus on early optimization of protein and energy intake, prioritization of nutrient density and promotion of human milk feeding. Targeted micronutrient support, individualized fortification and multidisciplinary care are essential to improve pulmonary and neurodevelopmental outcomes.

## 1. Introduction

Bronchopulmonary dysplasia (BPD) is the most prevalent chronic complication of prematurity, affecting approximately 40% of the extremely preterm (EPT) infants. This condition is a major risk factor for increased morbidity and mortality, with implications that extending beyond pulmonary morbidity to growth, neurodevelopment, and long-term health [1,2]. Its evolving pathophysiologic models emerge from a persistent cycle of inflammation, oxidative stress, structural injury, and reparative process in the lung’s alveolar and vascular compartments. It’s a multi-triggered by a combination of pre- and postnatal factors. The development of this condition is multifactorial, influenced by a combination of pre- and postnatal factors. Among the various postnatal factors, nutrition is recognized as playing a fundamental role in lung growth and repair [3,4].

Following a preterm birth, several issues linked to extreme immaturity make it difficult for these infants to get enough energy and nutrients. This can lead to nutritional deficiencies in the first few weeks of life, which contributes to ongoing lung injury and hinders alveolar development. Consequently, low postnatal growth rates are directly correlated with a higher risk of BPD [5,6]. Nutrition is considered one of the most important modifiable factors for preventing this condition.

For infants with established BPD, nutritional needs are substantially higher—an estimated 15–20% increase in caloric needs compared to infants with healthy lung development [7]. This is primarily due to the increased work of breathing, the metabolic demands associated with growth, and the energy required for tissue repair. Therefore, the nutritional management of BPD must focus on meeting these high demands to support somatic growth, tissue repair, and lung development [8]. Failure to do so often results in growth restriction, which can worsen the disease’s progression and further compromise lung and functional development, creating a vicious cycle. Addressing the interplay between increased metabolic demands, therapeutic constraints, and feeding difficulties is therefore essential to optimizing outcomes.

The close relationship between nutrition and BPD highlights why nutritional interventions are so critical for these vulnerable infants. While advances in neonatal care have improved survival rates for premature babies, BPD remains a significant challenge, with long-term consequences for a child’s health. The aim of this review is to provide a comprehensive analysis of the latest evidence on the role of nutrition in both preventing and managing BPD. We will explore specific nutritional strategies and their impact, with the goal of guiding clinical practice and improving outcomes for this high-risk population.

## 2. Literature Search

A comprehensive literature search was performed across major databases, including PubMed and Medline. Keywords such as “Premature infant”, “Very Low Birth Weight Infant”, “Preterm birth”, “Energy Intake”, “Nutrition”, “Nutrition Therapy”, “Premature Infant Diseases”, and “Bronchopulmonary Dysplasia” were used in various combinations, with the latest search conducted in August 2025. We identified and screened 2890 studies. Only studies published in English were included. In vitro and animal model studies were excluded. Additionally, references from relevant articles were screened to identify other pertinent studies not captured in the initial search. Recent articles were selected based on the authors’ judgment regarding their relevance to the topic.

## 3. Nutrition Strategies for the BPD Prevention

Adequate postnatal nutrition plays a fundamental role in maintaining growth and promoting lung repair in preterm infants and is recognized as one of the modifiable factors in the prevention of BPD. Nutritional deficiency, with inadequate calorie intake, in the first four weeks of life, especially in the first week, contributes to the persistence of lung injury and the failure of alveolar development and increases the risk of BPD [5,6,9,10,11].

## 4. Impact of Parenteral Nutrition

Most of the available evidence on this topic addresses both enteral nutrition (EN) and parenteral nutrition (PN). In particular, PN has been extensively investigated, and strategies related to its composition have been identified as potentially protective against the development of BPD.

## 5. Macronutrients and Total Energy Delivery

Inadequate postnatal nutrient intake has been consistently associated with an increased risk of BPD. Evidence suggests that higher protein and lipid provision during the early postnatal period exerts a protective effect. Protein intake up to 4 g/kg/day and lipid intake of 3–3.5 g/kg/day have been linked to a reduced incidence of BPD, with protein playing a particularly important role in somatic growth and neurodevelopment [12]. Similarly, a retrospective analysis of PN reported that amino acid intakes ≥ 1.5 g/kg/day during the first week of life were associated with shorter duration of mechanical ventilation (MV), decreased oxygen supplementation, and lower BPD incidence. In mechanically ventilated infants requiring support beyond 10 days, higher energy and protein intakes also conferred a reduced risk [13]. More recently, a multicenter study involving 40 neonatal centers in China highlighted the importance of careful titration. While PN should provide a balanced supply of energy and nutrients, excessive protein administration (>3.5 g/kg/day during the first week) was associated with an increased risk of BPD, underscoring the need to avoid over-supplementation [14].

The composition of intravenous lipid emulsions (IVLEs) has also been investigated in relation to BPD. New-generation formulations enriched with omega-3 fatty acids, such as SMOF lipid, have theoretical anti-inflammatory advantages. However, evidence remains inconclusive. While some retrospective studies reported reductions in BPD, severe retinopathy of prematurity, and cholestasis with SMOF lipid use [15], meta-analyses and prospective cohort studies found no significant association between newer emulsions and reductions in BPD or mortality [16,17]. Methodological heterogeneity likely contributes to these discrepancies, underscoring the need for further high-quality trials.

Total energy delivery has also been shown to influence BPD outcomes. An energy intake below 60 kcal/kg/day during the first week of life doubled the risk of BPD, while each additional 10 kcal/kg/day between days 7 and 27 was associated with a 9% reduction in risk [5,18]. Infants who developed severe BPD had significantly lower cumulative caloric intake during the first two weeks compared with those who did not [19]. Newborns who developed BPD received a calorie/protein ratio below that recommended for preterm newborns’ growth during the first two weeks of life, and this imbalance persisted until the fourth week in most cases [9]. These findings highlight the importance of early optimization of energy provision. The timing of PN initiation, however, remains a subject of debate. Some retrospective studies have associated early PN with higher BPD incidence, though this may reflect confounding by illness severity and increased survival among the most vulnerable infants [20,21]. Larger, prospective studies are needed to define optimal macronutrient targets and clarify the role of PN timing in BPD prevention.

Fluid management is another determinant of BPD risk, though the evidence is complex. Early fluid restriction has been suggested as protective, with a 2014 Cochrane review reporting a trend toward reduced BPD and other morbidities, albeit without statistical significance [22]. More recent studies, however, consistently associate high fluid intake with increased BPD severity. For example, infants with moderate to severe BPD had significantly higher daily intakes (150–180 mL/kg/day) during the first week of life, and prolonged MV has been linked to higher peak fluid balance [11,23]. Case–control data further suggest that cumulative fluid intake over the first two weeks is strongly associated with severe BPD [19]. In contrast, some studies have identified lower fluid intake as a risk factor, likely reflecting therapeutic restriction in infants with evolving BPD or patent ductus arteriosus [24]. Importantly, several analyses indicate that infants who develop BPD often receive fluids with a lower calorie-to-volume ratio, highlighting that inadequate nutrient density, rather than volume alone, may be critical.

Overall, the evidence suggests that strategies to mitigate BPD risk should prioritize early and adequate delivery of protein and energy, careful consideration of lipid emulsion composition, and judicious management of fluid balance. It is also essential to initiate early lipids to reach caloric goals. Initial fluid restriction may offer short-term protection, but once enteral feeding is established, advancing to higher volumes of nutrient-rich feeds appears both safe and essential for growth. This is supported by randomized trial evidence demonstrating that high enteral volumes (180–200 mL/kg/day) promote superior growth without increasing the incidence of BPD or other complications [25]. Despite these advances, significant gaps remain, and future large-scale, prospective trials are required to establish evidence-based nutritional and fluid management protocols for reducing BPD risk in preterm infants.

## 6. Impact of Enteral Nutrition and Human Milk

Early initiation and sustained provision of EN, particularly mother’s own milk (MOM), appear to exert a protective effect against BPD. Delayed initiation of EN beyond the third day of life has been associated with a 4.5-fold increased risk of BPD [26], a finding consistently supported in subsequent studies. MOM feeding within the first 72 h after birth, and its continuation throughout hospitalization, was associated with a lower incidence of moderate to severe BPD in very low birth weight (VLBW) infants [27]. Similarly, Uberos et al. [28] reported that preferential administration of MOM during the first two weeks of life significantly reduced the probability of both mild and severe BPD.

More recent evidence highlights the importance of cumulative exposure and timing of MOM. A retrospective study demonstrated that earlier initiation and greater overall intake of MOM were associated with a reduction in moderate to severe BPD, with benefits evident as early as the first week of life [29]. Importantly, the proportion of MOM provided throughout hospitalization appeared to mitigate the effect of gestational age on BPD severity. In another single-center observational study, preterm infants ≤ 1500 g birth weight who did not develop BPD had received significantly greater volumes of human milk (HM) during the first six weeks of life compared to those who developed BPD. Even after multivariate adjustment, HM intake remained inversely associated with BPD incidence [30].

A dose–response association had already been described by Patel et al., who found a 9.5% reduction in BPD odds for each 10% increase in MOM feedings up to 36 weeks postmenstrual age (PMA) [31]. Similarly, in EPT infants (22–26 weeks’ gestation), exclusive HM feeding and HM at discharge were associated with significantly lower rates of moderate to severe BPD [32].

Feeding advancement strategies may also influence respiratory outcomes. Yang et al. reported that infants with rapid feeding progression (≥30 mL/kg/day) were less likely to require mechanical ventilation, although this did not translate into a measurable reduction in BPD incidence [33]. Meanwhile, recent large multicenter study from China found that prolonged time to achieve full enteral nutrition in very preterm infants was independently associated with an increased risk of BPD [14].

The biological plausibility of these findings is supported by the unique properties of HM. Beyond providing adequate nutrients, HM contains antioxidants and bioactive factors with anti-inflammatory potential. Human milk oligosaccharides (HMOs) are metabolized by the intestinal microbiota into short-chain fatty acids (SCFAs), particularly acetic acid, which may play a protective role. Low fecal acetic acid levels on days 14 and 28 have been linked to increased risk of BPD, suggesting a critical interplay between intestinal health, the microbiome, and pulmonary outcomes in preterm infants [34].

### 6.1. Human Milk vs. Formula

Accumulating evidence indicates that HM confers a protective effect against BPD when compared with formula feeding. Two systematic reviews and meta-analyses conducted by Villamor-Martínez et al. demonstrated that exclusive MOM feeding was associated with a significant reduction in BPD risk, particularly when compared with mixed MOM plus any preterm formula (PF) diets [35]. An earlier review by the same group showed that donor human milk (DHM) supplementation was also protective against BPD and reduced days of mechanical ventilation when compared with PF; furthermore, an exclusive HM diet consistently reduced BPD incidence compared with PF-containing diets [36]. The evidence was predominantly derived from observational studies and limited by several factors, including high population heterogeneity, variable BPD definitions, and inconsistencies in intervention duration, which reduces the reliability of the results.

A subsequent meta-analysis confirmed that both exclusive and partial HM diets were associated with reduced BPD risk compared with exclusive formula feeding, though study heterogeneity and risk of bias temper the strength of these findings [37].

More recent retrospective studies provide additional support. Peng et al. reported that exclusive HM feeding reduced BPD incidence by 31% among very/extremely low birth weight infants, while also lowering the risks of necrotizing enterocolitis (NEC), severe retinopathy of prematurity (ROP), and mortality [38]. Similarly, in EPT infants (22–26 weeks’ gestation), Verd et al. observed that exclusive HM feeding reduced the odds of moderate-to-severe BPD by 25%, and receipt of any HM at discharge reduced the odds by 29% [32]. Together, these data reinforce the dose-dependent benefits of HM over formula in mitigating BPD risk.

### 6.2. Mother’s Own Milk vs. Donor Human Milk

Whether MOM offers superior protection against BPD compared with DHM remains uncertain. Villamor-Martínez et al. found no significant difference in BPD incidence between infants fed MOM versus DHM [36]. More recent studies have confirmed these findings: Merino-Hernández et al. and Avila-Álvarez both reported no differences in BPD rates between MOM-fed and DHM-fed preterm infants [39,40].

Descriptive data from Uberos et al. suggested that infants who developed BPD were less likely to have received DHM during the second week of life (9.3% vs. 21.8%), although this was not based on a direct MOM–DHM comparison [28]. Siddiqui et al., in a large retrospective study of infants < 34 weeks’ gestation and VLBW, also found no differences in BPD or other major prematurity-related outcomes between MOM- and DHM-fed groups [29]. Taken together, these studies suggest that DHM represents a safe and effective alternative when MOM is unavailable, without increasing the risk of BPD.

### 6.3. Fresh vs. Pasteurized HM

The impact of milk processing on BPD outcomes has also been investigated. Observational studies indicate significant advantages of fresh over pasteurized HM. A French multicenter study reported a 60% reduction in BPD risk among infants receiving fresh MOM, with no increase in NEC, sepsis, or mortality [41]. Similarly, found that very preterm infants fed fresh MOM had higher survival without severe morbidity, reduced BPD risk, faster achievement of full enteral feeds, earlier birth weight recovery, and shorter dependence on parenteral nutrition [42].

A systematic review and meta-analysis further confirmed the protective role of fresh versus pasteurized MOM, reporting a 23% reduction in BPD risk [36]. The evidence suggests that the pasteurization process may reduce the protective effect of human milk against BPD, due to the loss of bioactive components and antioxidants.

### 6.4. Use of Human Milk Fortification

Although HM is the optimal nutritional source, its inherent macro- and micronutrient content is insufficient to meet the accelerated growth demands of very preterm infants, underscoring the need for multi-nutrient fortification [43]. A Secondary analysis of a masked randomized controlled trial involving EPT infants revealed that early HM fortification (initiated on Day 3 versus Day 14 of life) was associated with a reduced severity of BPD at 36 weeks PMA. This effect may be mediated by modest increases in protein and energy intake, as well as improvements in length-for-age Z-scores, factors known to promote favorable lung development. However, the study did not demonstrate statistically significant differences in the rates of survival without BPD or predicted survival without BPD between the early and late fortification groups and it has some limitations as its limited statistical power.

A 2019 Cochrane review comparing human milk-derived and bovine milk-derived fortifiers in preterm infants exclusively fed HM found no statistically significant difference in the risk of BPD, although the certainty of the evidence was limited due to its derivation from a single study [44].

The use of fortified HM, utilizing either mother’s own milk or donor HM, particularly with early initiation, represents a strategy for achieving optimal nutritional goals and might mitigate the severity of BPD. The fortifiers increase the osmolality of the milk and risk of bacterial contamination, but there is no consistent data showing adverse effects on NEC [43]. Future research is warranted to corroborate these benefits and to further refine individualized nutritional strategies tailored to the specific needs of preterm infants.

## 7. Long-Chain Polyunsaturated Fatty Acids

Long-chain polyunsaturated fatty acids (LCPUFAs), particularly docosahexaenoic acid (DHA) and arachidonic acid (ARA), are essential for the development of preterm infants. DHA is a critical structural component of the brain and retina, supporting synaptogenesis, cortical development, and long-term neurodevelopmental outcomes. ARA, also abundant in the brain, is vital for neuronal signaling, cell membrane fluidity, and overall brain growth. DHA and ARA act synergistically, emphasizing the importance of maintaining a balanced ratio for optimal neurological development [45,46].

These fatty acids and their metabolites have been hypothesized to contribute to the repair and maturation of immature lungs, potentially preventing BPD. Observational studies have linked low postnatal DHA and ARA blood levels in preterm infants to higher risks of neonatal morbidities, including BPD [47,48].

A 2023 meta-analysis (4 studies) including 2,304 infants born before 29 weeks’ gestation evaluated high-dose oral DHA supplementation and BPD risk [49]. One of the studies, with the largest sample size and a DHA dose of 60 mg/kg/day, had a prominent influence on this meta-analysis, shaping the overall result [50]. Another important factor is the variation and inconsistencies in the DPB classification resulting in high heterogeneity in overall results. While the overall analysis did not demonstrate a significant association, a subgroup analysis—using BPD defined as supplemental oxygen or respiratory support at 36 weeks’ PMA—suggested an increased risk of BPD, including moderate-to-severe cases, in DHA-supplemented infants. Consequently, this high-dose DHA supplementation is not recommended for BPD prevention in this population.

Subsequent research investigated combined enteral supplementation of ARA and DHA. In a secondary analysis of a trial including 204 EPT infants, ARA:DHA supplementation was not associated with increased BPD or pulmonary morbidity. Interestingly, higher serum ARA levels during the first 28 days correlated with reduced BPD severity, suggesting a potential role for ARA in lung development and underscoring the need to determine optimal ARA:DHA ratios. This result must be interpreted carefully, because the study did not have enough power to make this conclusion [51].

The 2025 individual participant data meta-analysis employing standardized definitions of BPD provides important clarification regarding the safety profile of high-doses DHA supplementation in infants born before 29 weeks’ gestation. By harmonizing BPD definitions, specifically focusing on severe BPD, the analysis reduces the diagnostic variability that has confounded prior studies and meta-analyses. To isolate the effect of DHA, the study excluded combined interventions, precluding analysis of complex interventions. The meta-analysis found that high-dose DHA supplementation was associated with an increased risk of “any degree of BPD” but not severe BPD in infants born before 29 weeks’ gestation. Given that DHA did not impact severe BPD—the key outcome for long-term prognosis—and considering its potential neurodevelopmental benefits, further evaluation of its use may be warranted [52].

In summary, while adding ARA and DHA is considered a reliable method of ensuring adequate PUFA provision, routine DHA supplementation specifically for BPD prevention is not recommended. However, aiming for an ARA/DHA ratio ranging from 1:1 to 2:1 appears to be safe, with a 2:1 ratio suggested for potential neurodevelopmental benefits [43]. Continued long-term follow-up studies are essential to fully elucidate the effects of these findings on respiratory and neurodevelopmental outcomes in childhood, particularly regarding ARA:DHA ratios.

## 8. Vitamin Supplementation

Vitamins, acting as cofactors and regulators of metabolic and immune processes, modulate oxidative stress, epithelial integrity, and immune regulation—mechanisms central to BPD pathogenesis. Despite these potential benefits, evidence supporting their role in BPD prevention is limited, leading to inconsistent recommendations.

Vitamin A is the most studied micronutrient in this context. Intramuscular (IM) vitamin A supplementation has been associated with a modest reduction in BPD risk. Standard regimens involve 5,000 IU administered three times per week for 12–18 injections over four weeks. This intervention does not appear to significantly affect mortality, duration of mechanical ventilation, or incidence of NEC, although a modest improvement in oxygen-free days has been reported in some trials. Given that routine administration is not recommended, the decision to use repeated IM vitamin A doses for preventing chronic lung disease should be individualized, considering local disease incidence, potential benefit magnitude, lack of other proven benefits, treatment acceptance, and available alternatives. High-dose enteral vitamin A (5000 IU/kg/day) has proven ineffective, likely due to poor intestinal absorption in preterm infants [53,54].

A 2025 meta-analysis showed the potential protective effect of IM vitamin A, demonstrating a reduction in BPD risk, with regimens administered three times per week over 2–4 weeks yielding a significant decrease with moderate grade of certainty [55]. Despite this, the routine use of IM vitamin A is limited by the logistical challenges of multiple injections and the modest magnitude of benefit. Use should be individualized, considering local BPD incidence and feasibility, while systemic side effects remain rare.

Vitamin C exhibits antioxidant and anti-inflammatory properties; however, current evidence is insufficient to support its use for BPD prevention. The available meta-analytic data are of very low quality and carry a high risk of bias, which limits the reliability and the strength of any conclusions [55].

Similarly, vitamin D shows a potential, though uncertain, benefit in reducing BPD risk, with the certainty of evidence classified as very low due to limited studies and methodological concerns. Conversely, vitamin E has not demonstrated efficacy in preventing BPD, with meta-analytic data showing no significant effect [55].

Among vitamins, only intramuscular vitamin A shows modest efficacy in BPD prevention. Routine supplementation of vitamins C, D, or E is not supported. There is little evidence of long-term benefits, and oversupply, especially of fat-soluble vitamins, may have adverse effects. Any vitamin-based interventions should consider feasibility, potential adverse effects, and local clinical context.

## 9. Summary Nutrition Strategies for BPD Prevention

Adequate postnatal nutrition is a key modifiable factor in preventing BPD in preterm infants. Early deficits in calories and macronutrients, particularly protein (up to 4 g/kg/day) and lipids (3–3.5 g/kg/day), increase BPD risk, whereas higher energy intake in the first month is protective. Intravenous lipid emulsions may influence outcomes, but evidence remains inconsistent.

Fluid management is critical: high early fluid volumes and low calorie-to-volume ratios are associated with more severe BPD. Initial fluid restriction may be protective, but nutrient-rich enteral feeding at higher volumes supports growth without increasing BPD risk.

Enteral nutrition and human milk are strongly protective. Early and sustained MOM or DHM reduces BPD incidence and severity. Fresh HM may confer additional benefit compared to pasteurized milk.

DHA and ARA are essential for neurodevelopment and may aid lung maturation. High-dose DHA alone is not recommended for BPD prevention; optimal ARA:DHA ratios may provide combined pulmonary and neurological benefits. Vitamin supplementation shows limited efficacy. Intramuscular vitamin A modestly reduces BPD risk, while vitamins C, D, and E have no consistent benefit.

In summary, early optimization of energy and macronutrients, careful fluid and lipid management, timely enteral feeding, and human milk provision are central strategies to mitigate BPD risk in preterm infants.

## 10. Nutritional Strategies for Infants with Established BPD

Infants with BPD have markedly higher energy requirements than their peers without chronic lung disease, yet they often fail to achieve sufficient intake due to fluid restriction, feeding intolerance, respiratory compromise, or treatment-related side effects. The result is a characteristic pattern of poor growth, altered body composition, and delayed catch-up that can persist through infancy and even into childhood. Once BPD is established, nutritional management aims to support growth and optimize respiratory function while preventing long-term deficits, carefully respecting fluid and respiratory limitations.

## 11. Energy Demands, Growth, and Metabolic Challenges

Metabolic studies demonstrated early on that infants with severe respiratory distress or evolving BPD exhibit elevated resting energy expenditure, inefficient carbon dioxide elimination, and heightened oxygen consumption when exposed to increased glucose infusion [56]. These findings demonstrate the cumulative impact of increased work of breathing, recurrent hypoxemic episodes, and chronic systemic inflammation. This justifies the higher energy requirements observed in individuals with lung disease compared to their healthy peers, reflecting both respiratory and inflammatory demands [57]. This creates a persistent nutritional gap, particularly critical in the early postnatal period.

The consequences of this imbalance are well determined. Early cohorts revealed slower growth trajectories among infants with BPD compared to gestational age–matched control [58,59]. Persistent growth failure reflects the cumulative effects of premature birth, chronic lung disease, and prolonged exposure to intensive care interventions. Subsequent studies confirmed that suboptimal growth is multifactorial, driven by increased energy expenditure, nutrient deficits, and therapeutic side effects [1,60]. Body composition analyses revealed that these deficits persist beyond hospital discharge, with BPD infants showing lower fat-free mass and total fat during the first year of life [61]. Poor growth in this window carries critical neurodevelopmental implications, as impaired head circumference growth in the first year has been associated with subsequent cognitive and learning difficulties [62]. Furthermore, catch-up growth in children born with BPD is often delayed until late childhood, occurring between 8 and 14 years of age [61], partially explaining persisting deficits in school performance and motor outcomes [63]. Nutritional management in infants with BPD therefore requires balancing adequate growth with fluid and treatment limitations.

## 12. Feeding Difficulties and Nutritional Barriers

Feeding difficulties in BPD are common and multifactorial, including poor suck–swallow coordination, oral aversion after prolonged intubation, gastroesophageal reflux (GER), and frequent hypoxic episodes during feeding [64,65]. GER is common and often acid-predominant, particularly in extremely low birth weight infants. pH impedance studies show higher reflux event frequency and symptom sensitivity in BPD infants compared to preterm controls [66]. Although transpyloric feeding has been suggested as an alternative, randomized evidence failed to show reductions in hypoxemia in severe BPD [67]. Pharmacologic therapy should be reserved for confirmed pathological reflux due to safety concerns with acid suppression [66]. Alongside these feeding barriers, widely used therapies further complicate nutritional care. Diuretics induce urinary sodium and mineral losses [7], corticosteroids promote protein catabolism and fluid restriction, though often necessary, reduces caloric delivery and risks renal complications if not carefully monitored [2].

## 13. Nutritional Strategies and Interventions

PN is frequently required in the early phases of life, yet infants who later develop BPD often receive less energy in the first month than peers without lung disease [68]. For those at high risk, PN should supply 80–100 kcal/kg/day in the first week, advancing to 120–150 kcal/kg/day by weeks 2–4 [69]. Amino acid provision should begin as early as possible, with rapid escalation to 3.5 g/kg/day [70]. Lipid emulsions, especially those incorporating fish oil and medium-chain triglycerides, offer a dense energy source with lower carbon dioxide production than glucose, making them particularly suited for infants with compromised pulmonary function [71].

Once enteral nutrition is established, energy requirements often exceed those recommended for healthy preterm infants. While guidelines propose 110–130 kcal/kg/day for uncomplicated preterms [72], infants with BPD may need 130–150 kcal/kg/day, and in severe cases up to 150 kcal/kg/day, alongside protein intakes of 3.5–4 g/kg/day [2]. Protein has emerged as a critical limiting nutrient: greater protein/energy supply accelerates weight gain and improves lean mass accretion, lung tissue repair, and optimal growth [73]. Carbohydrates provide readily available energy but increase carbon dioxide production, potentially aggravating respiratory acidosis, whereas lipids offer a more favorable respiratory quotient. Theoretical benefits of high-fat regimens, however, have not translated into measurable improvements in pulmonary outcomes [74]. In practice, balanced macronutrient delivery—tailored to tolerance and growth—remains the most effective approach.

Human milk remains the optimal source of nutrition for preterm infants, offering immunological and neurodevelopmental benefits. Yet unfortified milk cannot meet the increased requirements of BPD infants, necessitating fortification. Individualized strategies based on milk analysis and biochemical monitoring improve growth without metabolic complications [73]. When mother’s milk is unavailable, nutrient-enriched preterm formulas provide higher energy and protein density and have been shown to improve linear growth, bone mineralization, and lean body mass compared with standard formulas [75,76]. Concentrating feeds above 80 kcal/100 mL with modular supplements is possible, though risks of osmolality-related intolerance or NEC demand caution [77].

Micronutrient supplementation is equally important. Iron deficiency is highly prevalent and requires supplementation from 4–8 weeks of life through the first year [1,64]. Zinc has been shown to improve growth in human milk–fed infants with BPD [78], and sodium supplementation is frequently necessary to counteract diuretic-associated losses. Chronic steroid and diuretic use, coupled with fluid restriction, predispose to metabolic bone disease, highlighting the importance of adequate calcium, phosphorus, and vitamin D [68,79]. Trace elements such as selenium and copper are also essential to support antioxidant defenses [79].

Feeding problems are so prevalent that many infants ultimately require tube feeding, and in severe cases gastrostomy, to secure adequate intake [1,64]. Early and sustained involvement of multidisciplinary teams—including neonatology, pulmonology, gastroenterology, dietetics, and feeding specialists—is critical to minimize complications and to develop individualized plans.

## 14. Post-Discharge Nutrition and Long-Term Outcomes

The nutritional vulnerability of infants with BPD extends beyond the neonatal intensive care unit. Growth restriction frequently persists after discharge, and nutritional status in the first two years predicts later pulmonary outcomes [75]. Post-discharge strategies must therefore ensure nutrient-enriched feedings until catch-up growth is achieved, with close monitoring of growth velocity, biochemical markers, and feeding tolerance [80,81].

Human milk fortification after discharge improves growth without increasing intolerance and supports higher exclusive breastfeeding rates compared with formula use. For exclusively breastfed infants with BPD who demonstrate inadequate growth, post-discharge formulas may be considered, either as a partial replacement for or an alternative to breastfeeding. These formulas are enriched with energy and nutrients, designed for short- to medium-term use in preterm infants after hospital discharge, and have a nutrient profile intermediate between preterm and standard term formulas [2].

If growth failure persists despite the use of a post-discharge formula, switching to a preterm formula may be warranted. Preterm formulas provide higher nutrient density per 100 mL, including energy (≈80 vs. 73 kcal), protein (2.4 vs. 1.9 g), calcium (≈140 vs. 80 mg), and phosphorus (≈75 vs. 50 mg). In a clinical trial, infants with BPD who received preterm formula demonstrated significantly greater length, bone mineral content, and lean mass at three months’ corrected age compared with those fed term formula [76].

While these formulas can effectively augment caloric intake, their use should be reserved as a last-line intervention. The introduction of formula feeding may negatively impact the sustainability of breastfeeding. The implementation of alternative strategies to optimize caloric and nutritional requirements should be prioritized before considering formula supplementation. A multidisciplinary approach is essential to ensure individualized nutritional management and optimize long-term outcomes in this high-risk population [1,64].

## 15. Summary Nutrition Strategies for Established BPD

In summary, nutrition in established BPD is a cornerstone of management. Elevated metabolic demands, restrictive therapies, and complex feeding barriers converge to create a high-risk scenario for growth failure and neurodevelopmental impairment. Optimal care requires aggressive but carefully balanced energy and protein delivery, fortified human milk or enriched formulas, comprehensive micronutrient support, and sustained nutritional follow-up after discharge. Future advances will depend on individualized strategies, integration of emerging tools such as milk composition analysis and body composition monitoring, and rigorous evaluation of how early nutritional practices influence long-term respiratory and neurodevelopmental trajectories.

## 16. Conclusions

Nutrition plays a pivotal role in both the prevention and management of bronchopulmonary dysplasia in preterm infants. Early postnatal deficits in energy, protein, and lipids increase BPD risk, while timely, adequate provision of macronutrients—through optimized parenteral and enteral strategies—supports lung growth, repair, and overall development. Human milk, particularly mother’s own milk, offers additional protective effects, and its early and sustained provision is strongly associated with reduced BPD incidence and severity. In established BPD, elevated metabolic demands, feeding difficulties, and therapeutic restrictions necessitate individualized nutritional strategies that prioritize energy and protein delivery, fortification, micronutrient supplementation, and long-term monitoring. While advances in nutritional care have improved outcomes, ongoing research is required to refine macronutrient targets, optimize fatty acid supplementation, and personalize feeding approaches to maximize pulmonary, growth, and neurodevelopmental outcomes in this high-risk population (Table 1).

## Figures and Tables

**Table 1:** Summary Board with Nutritional Key Recommendations for preventing and managing Bronchopulmonary Dysplasia.

Category	Key Recommendations
**Early Postnatal Nutrition**	Adequate calories and macronutrients are critical; deficits in the first 1–4 weeks increase BPD risk. Target protein: 3.5–4 g/kg/day, lipids: 3–3.5 g/kg/day. Energy intake < 60 kcal/kg in week 1 doubles BPD risk.
Parenteral Nutrition	Early PN can provide essential protein and energy. Amino acids ≥ 1.5 g/kg/day in week 1 reduce mechanical ventilation duration and BPD incidence. SMOF lipid have theoretical anti-inflammatory benefits, but evidence is inconclusive.
Fluid Management	Initial fluid restriction may help, but nutrient-rich enteral feeds at adequate volumes are essential once tolerated. High early fluid volumes and low calorie-to-volume ratios increase BPD severity.
Enteral Nutrition	Early initiation (within 72 h) and sustained provision of MOM or DHM reduces BPD incidence and severity. Fresh MOM may be superior to pasteurized milk.
HM vs. Formula	Exclusive human milk feeding reduces BPD risk compared to formula; benefits are dose-dependent. DHM is a safe alternative when MOM is unavailable.
LCPUFAs	DHA and ARA support neurodevelopment and may aid lung maturation. High-dose DHA alone is **not** recommended. ARA/DHA ratio 2:1 is recommended [43].
Vitamin Supplementation	Only intramuscular vitamin A shows modest BPD risk reduction. Vitamins C, D, E have no consistent benefit; oversupply may be harmful.
**Nutrition in Established BPD**	Infants require 15–25% higher energy. Protein 3.5–4 g/kg/day, energy 130–150 kcal/kg/day (up to 150 kcal/kg/day in severe cases). Fortified human milk or enriched formulas are preferred.
Micronutrients	Iron, zinc, sodium, calcium, phosphorus, vitamin D, selenium, and copper are important to support growth, bone health, and antioxidant defenses.
Feeding Challenges	Poor suck–swallow coordination, GER, oral aversion, and respiratory compromise often require tube feeding or gastrostomy. Multidisciplinary support is essential.
Post-Discharge Nutrition	Nutrient-enriched feedings should continue until catch-up growth. Close monitoring of growth, biochemical markers, and feeding tolerance is critical.

## Data Availability

The original contributions presented in the paper are included in the review article. Further inquiries can be directed to the corresponding author.

## Abbreviations

The following abbreviations are used in this manuscript:

ARA	Arachidonic Acid
DHA	Docosahexaenoic Acid
DHM	Donor Human Milk
EN	Enteral Nutrition
EPT	Extremely Preterm
HM	Human Milk
HMOs	Human milk oligosaccharides
IVLEs	Intravenous Lipid Emulsions
LCPUFAs	Long-chain polyunsaturated fatty acids
MOM	Mother’s Own Milk
MV	Mechanical Ventilation
NEC	Necrotizing Enterocolitis
PF	Preterm Formula
PMA	Postmenstrual Age
PN	Parenteral Nutrition
ROP	Retinopathy of Prematurity
SCFAs	Short-Chain Fatty Acids
SMOF	soya-bean oil, medium-chain triglycerides, olive oil and fish oil
VLBW	Very Low Birth Weight

## References

[1] Poindexter B.B., Martin C.R. (2015). Impact of Nutrition on Bronchopulmonary Dysplasia. Clin. Perinatol..

[2] Rocha G., Guimarães H., Pereira-Da-silva L. (2021). The role of nutrition in the prevention and management of bronchopulmonary dysplasia: A literature review and clinical approach. Int. J. Environ. Res. Public Health.

[3] Bancalari E., Jain D. (2019). Bronchopulmonary Dysplasia: 50 Years after the Original Description. Neonatology.

[4] Ambalavanan N., Deutsch G., Pryhuber G., Travers C.P., Willis K.A. (2026). The evolving pathophysiology of bronchopulmonary dysplasia. Physiol. Rev..

[5] Uberos J., Jimenez-Montilla S., Molina-Oya M., García-Serrano J.L. (2020). Early energy restriction in premature infants and bronchopulmonary dysplasia: A cohort study. Br. J. Nutr..

[6] Gilfillan M., Bhandari A., Bhandari V. (2021). Diagnosis and management of bronchopulmonary dysplasia. BMJ.

[7] Carlson S.J. (2004). Invited Review Current Nutrition Management of Infants with Chronic Lung Disease. Nutr. Clin. Pract..

[8] Hwang J.S., Rehan V.K. (2018). Recent Advances in Bronchopulmonary Dysplasia: Pathophysiology, Prevention, and Treatment. Lung.

[9] Milanesi B.G., Lima P.A., Villela L.D., Martins A.S., Gomes-Junior S.C.S., Moreira M.E.L., Méio M.D.B.B. (2021). Assessment of early nutritional intake in preterm infants with bronchopulmonary dysplasia: A cohort study. Eur. J. Pediatr..

[10] Thiess T., Lauer T., Woesler A., Neusius J., Stehle S., Zimmer K.-P., Eckert G.P., Ehrhardt H. (2021). Correlation of Early Nutritional Supply and Development of Bronchopulmonary Dysplasia in Preterm Infants <1,000 g. Front. Pediatr..

[11] Al-Jebawi Y., Argawal N., Groh Wargo S., Shekhawat P., Mhanna M.J. (2020). Low caloric intake and high fluid intake during the first week of life are associated with the severity of bronchopulmonary dysplasia in extremely low birth weight infants. J. Neonatal Perinat. Med..

[12] Panagiotounakou P., Sokou R., Gounari E., Konstantinidi A., Antonogeorgos G., Grivea I.N., Daniil Z., Gourgouliannis K.I., Gounaris A. (2019). Very preterm neonates receiving “aggressive” nutrition and early nCPAP had similar long-term respiratory outcomes as term neonates. Pediatr. Res..

[13] Yang J., Chang S.S.Y., Poon W.B. (2016). Relationship between Amino Acid and Energy Intake and Long-Term Growth and Neurodevelopmental Outcomes in Very Low Birth Weight Infants. J. Parenter. Enter. Nutr..

[14] Lin H., Bai G., Ge J., Chen X., He X., Ma X., Shi L., Du L., Chen Z. (2024). Nutritional support during the first week for infants with bronchopulmonary dysplasia and respiratory distress: A multicenter cohort study in China. BMC Pediatr..

[15] Wang Y.L., Chen L.J., Tsao L.Y., Chen H.N., Lee C.H., Hsiao C.C. (2021). Parenteral nutrition with fish oil-based lipid emulsion reduces the risk of cholestasis in preterm infants. J. Int. Med. Res..

[16] Ndiaye A.B., Mohamed I., Pronovost E., Angoa G., Piedboeuf B., Lemyre B., Afifi J., Qureshi M., Sériès T., Guillot M. (2022). Use of SMOF lipid emulsion in very preterm infants does not affect the incidence of bronchopulmonary dysplasia–free survival. J. Parenter. Enter. Nutr..

[17] Fan X., Tang Y., Tang J., Chen J., Shi J., Wang H., Xia B., Qu Y., Mu D. (2020). New-generation intravenous fat emulsions and bronchopulmonary dysplasia in preterm infants: A systematic review and meta-analysis. J. Perinatol..

[18] Klevebro S., Westin V., Sjöström E.S., Norman M., Domellöf M., Bonamy A.-K.E., Hallberg B. (2019). Early energy and protein intakes and associations with growth, BPD, and ROP in extremely preterm infants. Clin. Nutr..

[19] Kolitz D., Przystac L., Tucker R., Oh W., Stonestreet B.S. (2024). Higher fluid and lower caloric intakes: Associated risk of severe bronchopulmonary dysplasia in ELBW infants. J. Perinatol..

[20] Webbe J.W.H., Longford N., Battersby C., Oughham K., Uthaya S.N., Modi N., Gale C. (2022). Outcomes in relation to early parenteral nutrition use in preterm neonates born between 30 and 33 weeks’ gestation: A propensity score matched observational study. Arch. Dis. Child. Fetal Neonatal Ed..

[21] Uthaya S., Longford N., Battersby C., Oughham K., Lanoue J., Modi N. (2022). Early versus later initiation of parenteral nutrition for very preterm infants: A propensity score-matched observational study. Arch. Dis. Child. Fetal Neonatal Ed..

[22] Bell E.F., Acarregui M.J. (2014). Restricted versus liberal water intake for preventing morbidity and mortality in preterm infants. Cochrane Database Syst. Rev..

[23] Starr M.C., Griffin R., Gist K.M., Segar J.L., Raina R., Guillet R., Nesargi S., Menon S., Anderson N., Askenazi D.J. (2022). Association of Fluid Balance with Short- and Long-term Respiratory Outcomes in Extremely Premature Neonates: A Secondary Analysis of a Randomized Clinical Trial. JAMA Netw. Open.

[24] Malikiwi A.I., Lee Y.M., Davies-Tuck M., Wong F.Y. (2019). Postnatal nutritional deficit is an independent predictor of bronchopulmonary dysplasia among extremely premature infants born at or less than 28 weeks gestation. Early Hum. Dev..

[25] Travers C.P., Wang T., Salas A.A., Schofield E., Dills M., Laney D., Yee A., Bhatia A., Winter L., Ambalavanan N. (2020). Higher- or Usual-Volume Feedings in Infants Born Very Preterm: A Randomized Clinical Trial. J. Pediatr..

[26] Konnikova Y., Zaman M.M., Makda M., D’Onofrio D., Freedman S.D., Martin C.R. (2015). Late enteral feedings are associated with intestinal inflammation and adverse neonatal outcomes. PLoS ONE.

[27] Zhu Y., Chen X., Zhu J., Jiang C., Yu Z., Su A. (2022). Effect of First Mother’s Own Milk Feeding Time on the Risk of Moderate and Severe Bronchopulmonary Dysplasia in Infants With Very Low Birth Weight. Front. Pediatr..

[28] Uberos J., Sanchez-Ruiz I., Fernández-Marin E., Ruiz-López A., Cubero-Millan I., Campos-Martínez A. (2024). Breast-feeding as protective factor against bronchopulmonary dysplasia in preterm infants. Br. J. Nutr..

[29] Siddiqui A., Voynow J., Chahin N., Williams A., Xu J., Chavez D., Carroll L., Hendricks-Muñoz K.D. (2025). Greater and Earlier Exposure of Mother’s Own Milk Compared to Donor Human Milk Moderates Risk and Severity of Bronchopulmonary Dysplasia. Breastfeed. Med..

[30] Fonseca L.T., Senna D.C., Silveira R.C., Procianoy R.S. (2017). Association between Breast Milk and Bronchopulmonary Dysplasia: A Single Center Observational Study. Am. J. Perinatol..

[31] Patel A.L., Johnson T.J., Robin B., Bigger H.R., Buchanan A., Christian E., Nandhan V., Shroff A., Schoeny M., Engstrom J.L. (2017). Influence of own mother’s milk on bronchopulmonary dysplasia and costs. Arch. Dis. Child. Fetal Neonatal Ed..

[32] Verd S., Porta R., Ginovart G., Avila-Alvarez A., Rodrigo F.G.-M., Renau M.I., Ventura P.S. (2023). Human Milk Feeding Is Associated with Decreased Incidence of Moderate-Severe Bronchopulmonary Dysplasia in Extremely Preterm Infants. Children.

[33] Yang W.-C., Fogel A., Lauria M.E., Ferguson K., Smith E.R. (2022). Fast Feed Advancement for Preterm and Low Birth Weight Infants: A Systematic Review and Meta-analysis. Pediatrics.

[34] Frazer L.C., Yakah W., Martin C.R. (2022). Decreased Acetic Acid in the Stool of Preterm Infants Is Associated with an Increased Risk of Bronchopulmonary Dysplasia. Nutrients.

[35] Villamor-Martínez E., Pierro M., Cavallaro G., Mosca F., Villamor E. (2019). Mother’s own milk and bronchopulmonary dysplasia: A systematic review and meta-analysis. Front. Pediatr..

[36] Villamor-Martínez E., Pierro M., Cavallaro G., Mosca F., Kramer B.W., Villamor E. (2018). Donor human milk protects against bronchopulmonary dysplasia: A systematic review and meta-analysis. Nutrients.

[37] Huang J., Zhang L., Tang J., Shi J., Qu Y., Xiong T., Mu D. (2019). Human milk as a protective factor for bronchopulmonary dysplasia: A systematic review and meta-analysis. Arch. Dis. Child. Fetal Neonatal Ed..

[38] Peng W., Han J., Li S., Zhang L., Yang C., Guo J., Cao Y. (2022). The Association of Human Milk Feeding With Short-Term Health Outcomes Among Chinese Very/Extremely Low Birth Weight Infants. J. Hum. Lact..

[39] Merino-Hernández A., Palacios-Bermejo A., Ramos-Navarro C., Caballero-Martín S., González-Pacheco N., Rodríguez-Corrales E., de Orgaz M.C.S.-G., Sánchez-Luna M. (2024). Effect of Donated Premature Milk in the Prevention of Bronchopulmonary Dysplasia. Nutrients.

[40] Avila-Alvarez A., Fernandez-Gonzalez S.M., Sucasas-Alonso A., Ansede A.S. (2025). Initiation of Enteral Feeding with Mother’s Own Milk or Donor Human Milk in Very Preterm Infants: Impact on Bronchopulmonary Dysplasia and Other Prematurity-Related Morbidities. Nutrients.

[41] Dicky O., Ehlinger V., Montjaux N., Gremmo-Féger G., Sizun J., Rozé J., Arnaud C., Casper C. (2017). Policy of feeding very preterm infants with their mother’s own fresh expressed milk was associated with a reduced risk of bronchopulmonary dysplasia. Acta Paediatr..

[42] Huang J., Zheng Z., Zhao X., Huang L., Wang L., Zhang X., Lin X. (2023). Short-term effects of fresh mother’s own milk in very preterm infants. Matern. Child Nutr..

[43] Embleton N.D., Jennifer Moltu S., Lapillonne A., van den Akker C.H.P., Carnielli V., Fusch C., Gerasimidis K., van Goudoever J.B., Haiden N., Iacobelli S. (2023). Enteral Nutrition in Preterm Infants (2022): A Position Paper from the ESPGHAN Committee on Nutrition and Invited Experts. J. Pediatr. Gastroenterol. Nutr..

[44] Premkumar M.H., Pammi M., Suresh G. (2019). Human milk-derived fortifier versus bovine milk-derived fortifier for prevention of mortality and morbidity in preterm neonates. Cochrane Database Syst. Rev..

[45] Carlson S.E., Colombo J. (2016). Docosahexaenoic Acid and Arachidonic Acid Nutrition in Early Development. Adv. Pediatr..

[46] Moltu S.J., Nordvik T., Rossholt M.E., Wendel K., Chawla M., Server A., Gunnarsdottir G., Pripp A.H., Domellöf M., Bratlie M. (2024). Arachidonic and docosahexaenoic acid supplementation and brain maturation in preterm infants; a double blind RCT. Clin. Nutr..

[47] Martin C.R., DaSilva D.A., Cluette-Brown J.E., DiMonda C., Hamill A., Bhutta A.Q., Coronel E., Wilschanski M., Stephens A.J., Driscoll D.F. (2011). Decreased postnatal docosahexaenoic and arachidonic acid blood levels in premature infants are associated with neonatal morbidities. J. Pediatr..

[48] Manley B.J., Makrides M., Collins C.T., McPhee A.J., Gibson R.A., Ryan P., Sullivan T.R., Davis P.G., for the DINO Steering Committee (2011). High-Dose Docosahexaenoic Acid Supplementation of Preterm Infants: Respiratory and Allergy Outcomes. Pediatrics.

[49] Marc I., Boutin A., Pronovost E., Herrera N.M.P., Guillot M., Bergeron F., Moore L., Sullivan T.R., Lavoie P.M., Makrides M. (2023). Association between Enteral Supplementation with High-Dose Docosahexaenoic Acid and Risk of Bronchopulmonary Dysplasia in Preterm Infants: A Systematic Review and Meta-analysis. JAMA Netw. Open.

[50] Collins C.T., Makrides M., McPhee A.J., Sullivan T.R., Davis P.G., Thio M., Simmer K., Rajadurai V.S., Travadi J., Berry M.J. (2017). Docosahexaenoic Acid and Bronchopulmonary Dysplasia in Preterm Infants. New Engl. J. Med..

[51] Wackernagel D., Nilsson A.K., Sjöbom U., Hellström A., Klevebro S., Hansen-Pupp I. (2024). Enteral supplementation with arachidonic and docosahexaenoic acid and pulmonary outcome in extremely preterm infants. Prostaglandins Leukot. Essent. Fat. Acids.

[52] Marc I., Lavoie P.M., Sullivan T.R., Pronovost E., Boutin A., Beltempo M., Guillot M., Gould J.F., Simonyan D., McPhee A.J. (2025). High-dose docosahexaenoic acid for bronchopulmonary dysplasia severity in very preterm infants: A collaborative individual participant data meta-analysis. Am. J. Clin. Nutr..

[53] Meyer S., Bay J., Franz A.R., Ehrhardt H., Klein L., Petzinger J., Binder C., Kirschenhofer S., Stein A., Hüning B. (2024). Early postnatal high-dose fat-soluble enteral vitamin A supplementation for moderate or severe bronchopulmonary dysplasia or death in extremely low birthweight infants (NeoVitaA): A multicentre, randomised, parallel-group, double-blind, placebo-controlled, investigator-initiated phase 3 trial. Lancet Respir. Med..

[54] Darlow B.A., Graham P.J., Rojas-Reyes M.X. (2016). Vitamin A supplementation to prevent mortality and short- and long-term morbidity in very low birth weight infants. Cochrane Database Syst. Rev..

[55] Bronnert A., Bloomfield P.M., Páramo L.D., Lin L., Bloomfield F.H., Cormack B.E. (2025). The effect of vitamin supplementation on neurodevelopmental and clinical outcomes in very low birth weight and very preterm infants: A systematic review and meta-analysis. PLoS ONE.

[56] De Meer K., Westerterp K.R., Houwen R.H.J., Brouwers H.A.A., Berger R., Okken A. (1997). Total energy expenditure in infants with bronchopulmonary dysplasia is associated with respiratory status. Eur. J. Pediatr..

[57] Principi N., Di Pietro G.M., Esposito S. (2018). Bronchopulmonary dysplasia: Clinical aspects and preventive and therapeutic strategies. J. Transl. Med..

[58] Davidson S., Schrayer A., Wielunsky E., Krikler R., Lilos P., Reisner S.H. (1990). Energy Intake, Growth, and Development in Ventilated Very-Low-Birth-Weight Infants with and Without Bronchopulmonary Dysplasia. Am. J. Dis. Child..

[59] Markestad T., Fitzhardinge P.M. (1981). Growth and Development in Children recovering from Bronchopulmonary Dysplasia. J. Pediatr..

[60] Bauer S.E., Huff K.A., Vanderpool C.P.B., Rose R.S., Cristea A.I. (2022). Growth and nutrition in children with established bronchopulmonary dysplasia: A review of the literature. Nutr. Clin. Pract..

[61] Huysman W.A., De Ridder M., De Bruin N.C., Van Helmond G., Terpstra N., Van Goudoever J.B., Sauer P.J.J. (2003). Growth and body composition in preterm infants with bronchopulmonary dysplasia. Arch. Dis. Child. Fetal. Neonatal Ed..

[62] Stathis S.L., O’Callaghan M., Harvey J., Rogers Y. (1999). Head circumference in ELBW babies is associated with learning difficulties and cognition but not ADHD in the school—Aged child. Dev. Med. Child. Neurol..

[63] Majnemer A., Riley P., Shevell M., Birnbaum R., Greenstone H., Coates A.L. (2000). Severe bronchopulmonary dysplasia increases risk for later neurological and motor sequelae in preterm survivors. Dev. Med. Child. Neurol..

[64] Biniwale M.A., Ehrenkranz R.A. (2006). The Role of Nutrition in the Prevention and Management of Bronchopulmonary Dysplasia. Semin. Perinatol..

[65] Jadcherla S.R. (2012). Pathophysiology of aerodigestive pulmonary disorders in the neonate. Clin. Perinatol..

[66] Nobile S., Noviello C., Cobellis G., Carnielli V.P. (2015). Are Infants with Bronchopulmonary Dysplasia Prone to Gastroesophageal Reflux? A Prospective Observational Study with Esophageal pH-Impedance Monitoring. J. Pediatr..

[67] Jensen E.A., Dysart K., Gantz M.G., McDonald S., Bamat N.A., Keszler M., Kirpalani H., Laughon M.M., Poindexter B.B., Duncan A.F. (2019). The Diagnosis of Bronchopulmonary Dysplasia in Very Preterm Infants An Evidence-based Approach. Am. J. Respir. Crit. Care Med..

[68] Gaio P., Verlato G., Daverio M., Cavicchiolo M.E., Nardo D., Pasinato A., de Terlizzi F., Baraldi E. (2018). Incidence of metabolic bone disease in preterm infants of birth weight <1250 g and in those suffering from bronchopulmonary dysplasia. Clin. Nutr. ESPEN.

[69] Joosten K., Embleton N., Yan W., Senterre T., The ESPGHAN/ESPEN/ESPR/CSPEN Working Group on Pediatric Parenteral Nutrition (2018). ESPGHAN/ESPEN/ESPR/CSPEN guidelines on pediatric parenteral nutrition: Energy. Clin. Nutr..

[70] van Goudoever J.B., Carnielli V., Darmaun D., de Pipaon M.S., The ESPGHAN/ESPEN/ESPR/CSPEN Working Group on Pediatric Parenteral Nutrition (2018). ESPGHAN/ESPEN/ESPR/CSPEN guidelines on pediatric parenteral nutrition: Amino acids. Clin. Nutr..

[71] Lapillonne A., Mis N.F., Goulet O., van den Akker C.H.V.D., Wu J., Koletzko B., Braegger C., Bronsky J., Cai W., Campoy C. (2018). ESPGHAN/ESPEN/ESPR/CSPEN guidelines on pediatric parenteral nutrition: Lipids. Clin. Nutr..

[72] Agostoni C., Buonocore G., Carnielli V.P., De Curtis M., Darmaun D., Decsi T., Domellöf M., Embleton N.D., Fusch C., Genzel-Boroviczeny O. (2010). Enteral nutrient supply for preterm infants: Commentary from the european society of paediatric gastroenterology, hepatology and nutrition committee on nutrition. J. Pediatr. Gastroenterol. Nutr..

[73] Giannì M.L., Roggero P., Colnaghi M.R., Piemontese P., Amato O., Orsi A., Morlacchi L., Mosca F. (2014). The role of nutrition in promoting growth in pre-term infants with bronchopulmonary dysplasia: A prospective non-randomised interventional cohort study. BMC Pediatr..

[74] Allen J., Zwerdling R., Ehrenkranz R., Gaultier C., Geggel R., Greenough A., Kleinman R., Klijanowicz A., Martinez F., Ozdemir A. (2003). Statement on the care of the child with chronic lung disease of infancy and childhood. Am. J. Respir. Crit. Care Med..

[75] Guimarães H., Rocha G., Guedes M.B., Guerra P., Silva A.I., Pissarra S. (2014). Nutrition of preterm infants with bronchopulmonary dysplasia after hospital discharge—Part II. J. Pediatr. Neonatal Individ. Med..

[76] Brunton J., Saigal S., Atkinson S.A. (1997). Growth and body composition in infants with bronchopulmonary dysplasia up to 3 months corrected age: A randomized trial of a high energy nutrient-enriched formula fed after hospital discharge. J. Pediatr..

[77] Lista G., Meneghin F., Bresesti I., Cavigioli F. (2017). Nutritional problems of children with bronchopulmonary dysplasia after hospital discharge. Pediatr. Medica E Chirurgica..

[78] Shaikhkhalil A.K., Curtiss J., Puthoff T.D., Valentine C.J. (2014). Enteral zinc supplementation and growth in extremely-low-birth-weight infants with chronic lung disease. J. Pediatr. Gastroenterol. Nutr..

[79] Pereira-Da-Silva L., Virella D., Fusch C. (2019). Nutritional assessment in preterm infants: A Practical Approach in the NICU. Nutrients.

[80] Bauer S.E., Vanderpool C.P.B., Ren C., Cristea A.I. (2021). Nutrition and growth in infants with established bronchopulmonary dysplasia. Pediatr. Pulmonol..

[81] Heras A., Chambers R., Solomon Z., Blatt L., Martin C.R. (2023). Nutrition-based implications and therapeutics in the development and recovery of bronchopulmonary dysplasia. Semin. Perinatol..

